# Short and Long-Term Effects of Anesthesia in *Octopus maya* (Cephalopoda, Octopodidae) Juveniles

**DOI:** 10.3389/fphys.2020.00697

**Published:** 2020-06-30

**Authors:** Katina Roumbedakis, Marina N. Alexandre, José A. Puch, Maurício L. Martins, Cristina Pascual, Carlos Rosas

**Affiliations:** ^1^AQUOS – Aquatic Organisms Health Laboratory, Department of Aquaculture, Federal University of Santa Catarina (UFSC), Florianopolis, Brazil; ^2^Unidad Mulidisicplinaria de Docencia e Investigación, Facultad de Ciencias, Universdidad Nacional Autónoma de México (UNAM), Mexico City, Mexico

**Keywords:** anesthetic, cephalopods, oxygen consumption, criteria, growth, mortality, animal welfare

## Abstract

This study aimed to explore different substances (or cold sea water) as potential anesthetic agents to facilitate short-term handling in *Octopus maya* juveniles. We investigated oxygen consumption before (baseline), during (first 600 s of exposure) and after anesthesia (recovery) of octopuses (*n* = 98; 1.67 ± 0.5 g) exposed to cold sea water (SW; 11 and 13°C), ethanol (EtOH; 0.5; 1.5 and 3.0%), magnesium chloride (MgCl_2_; 0.75; 1.5 and 3.75%), ethanol combined with magnesium chloride (Mix; 1.5:0.75%; 0.75:1.13%; and 2.25:0.37%) and clove oil (0.15 mL L^–1^). After exposure, the animals were handled for 180 s (exposed to air) and weighted. Two experimental groups not exposed to anesthetics (with or without handling) were also evaluated. The criteria for general anesthesia were analysed. Times of induction and recovery, incidence of attack response after recovery and possible longer-term effects of repeated general anesthesia on growth and mortality of the octopuses were evaluated. During anesthesia, *O. maya* juveniles exposed to SW (11 and 13°C), EtOH (0.5; 1.5 and 3.0%), Mix (0.75:1.13%), and clove oil, presented a significant decrease on oxygen consumption. In animals exposed to different concentrations of EtOH and Mix 0.75:1.13%, this decrease was registered after an increase on oxygen consumption. Animals exposed to MgCl_2_ did not show significant changes on oxygen consumption, except for animals exposed MgCl_2_ 3.75%, which showed a significant increase on oxygen consumption. At the end of recovery, except for octopuses exposed to clove oil and MgCl_2_ 0.75%, the values of oxygen consumption observed were comparable to the ones registered during baseline. Animals exposed to SW 11°C, EtOH 3.0%, Mix 1.5:0.75% and MgCl_2_ 3.75% fulfilled the criteria defined for general anesthesia. Exposure to MgCl_2_ (all concentrations), SW 13°C and clove oil reduced or inhibited the incidence of attack response after recovery. Except for animals exposed to clove oil, growth of the juveniles was not affected by the exposure to the different substances. Short-term handling (180 s) of *O. maya* juveniles can eventually be carried out without anesthesia. However, to facilitated handling, we suggest the use of EtOH 3.0% or cold sea water 11°C.

## Introduction

General anesthesia may be defined as a controllable and reversible state, which includes loss of consciousness, analgesia, suppression of reflex activity, and muscle relaxation (Flecknell, 2015). In aquatic animals, general anesthesia is commonly used for husbandry (e.g., handling, transport, and artificial reproduction), veterinary examination (e.g., blood sampling and pathological analysis), and for research purposes (e.g., to perform experimental procedures and surgery, facilitate manipulation, prevent injuries, reduce stress, and promote animal welfare). Regarding cephalopods, the topic has gained special attention in recent years, since their inclusion in the European legislation Directive 2010/63/EU on the protection of animals used for scientific purposes (European Parliament Council of the European Union, 2010).

Surprisingly, despite the recent increased interest, current information is available only for a few species using a limited variety of substances potentially acting as anesthetic agents: only about 20 substances have been investigated to anesthetize cephalopods (reviewed by Sykes et al., 2012; Gleadall, 2013b; Fiorito et al., 2015). Moreover, with few exceptions (Andrews and Tansey, 1981; Pugliese et al., 2016; Butler-Struben et al., 2018) the majority of anesthetic studies in cephalopods do not explore in-depth the efficacy of the commonly used agents and the physiological effects of anesthesia on the animals. Another limitation is the lack of standardized anesthesia protocols for cephalopods, since distinct methods are described in the literature.

Cephalopods are highly specialized invertebrates with particular characteristics, such as sophisticated brains and nervous systems (Shigeno et al., 2018) visual, mechanoreceptive, olfactory, and chemosensory abilities (Cooke et al., 2019b) complex behavioral and learning capabilities (Hanlon and Messenger, 2018) among others. These characteristics are important to improve the current understanding of cephalopods welfare requirements. In this sense, Ponte et al. (2019) provided a list of biological features that should be considered while assessing cephalopod welfare. From this list, three aspects are, in our view, particularly relevant in the context of cephalopod anesthesia: (i) respiration, including possible limitations that may affect the animals; (ii) inter-individual differences in behavioral repertoire and responses; and (iii) responses to stressors and negative experiences.

Physiological (e.g., respiration and heart rate) and physical aspects (e.g., skin color and body morphology) as well as behavioral responses (e.g., swimming activity, coordination, and inking) are utilized to evaluate and monitor anesthesia in cephalopods (Andrews and Tansey, 1981; Gonçalves et al., 2012; Gleadall, 2013b; Butler-Struben et al., 2018). Regarding physiological aspects, monitoring the respiration is a common practice during cephalopod anesthesia: reduction and even cessation of respiration have been utilized as criteria for anesthesia (Gleadall, 2013b; Fiorito et al., 2015) although increased respiration have been eventually observed during anesthesia induction (Andrews and Tansey, 1981; Messenger et al., 1985; Gonçalves et al., 2012). Nevertheless, potential physiological effects caused by oxygen consumption changes in cephalopods exposed to anesthetics have never been explored.

The effectiveness of anesthetics in relation to individual characteristics, including biological aspects (e.g., age, life stage, and sex), physiological condition and health status, is a gap in knowledge of cephalopod anesthesia (O’Brien et al., 2018). Inter-individual behavioral responses of cephalopods to anesthetics may differ depending on the species/taxon and the substance used (Butler-Struben et al., 2018). As observed in fishes (Readman et al., 2017) the responses to different anesthetic agents seem to be specie-specifics also in cephalopods (e.g., Andrews and Tansey, 1981; Gleadall, 2013b; Butler-Struben et al., 2018). Possible intra-specific differences may also occur depending on the life cycle stage and size/age of the animals or due to environmental variables (Sykes et al., 2012). Therefore, investigate different anesthetic agents and concentrations in the most commonly used cephalopod species for research and aquaculture purposes is fundamental to allow adequate handling and to ensure animal welfare to the highest standards.

The exposure to a stressor might cause short and long-term consequences in aquatic animals, affecting physiological and behavioral aspects, for example, directly impairing food intake, growth, reproduction, or other aspects of the normal performance (Ross and Ross, 2008). The purpose of use of anesthesia in animal experimentation is to contribute to improve animal welfare, however, some aspects should be considered. Before, during and after anesthesia, cephalopods can be exposed to potentially stressful situations, such as non-desirable side-effects due to exposure to substances as putative anesthetics and inadequate handling of the animals (Fiorito et al., 2015). Physiological consequences and eventual long-term effects due to the exposure of the animals to a stressor might occur, especially if the exposure is repeated. Food intake as well as body weight have been recommended as physiological indicators of cephalopod welfare, and, therefore, altered feeding behavior and/or reduction of body weight are considered objective measures of health and welfare status (for details, see Table 5C in Fiorito et al., 2015).

Within the scope of the Directive 2010/63/EU, animal research workers should reduce discomfort, pain, and suffering of animals used for research to an absolute minimum. In addition, it is legally required to assign the prospective severity classification of scientific procedures on live animals, as well as reporting the actual severity experienced by each animal used during the course of such procedures. To assist and guide cephalopod scientists in assessing severity classification of procedures using live cephalopods for research purposes, Cooke et al. (2019a) evaluated 50 scenarios covering different types of procedures. From these scenarios, 12 included the use (or lack) of anesthesia for different purposes, such as handling for growth rate comparisons, hemolymph sampling, surgery, among others.

During aquaculture and research practices, handling is commonly used for many purposes (e.g., selection, weighting, transport, management of broodstock, etc.). While handing cephalopods, a few aspects should be considered. For instance, their delicate skin can be eventually harmed (particularly relevant for squids and cuttlefishes) when removed from the water (Fiorito et al., 2014). A part from physical damage, stress caused by inadequate handling can promote a suppression of the immune response; in both cases, opportunistic secondary infections can eventually occur afterward (Sykes et al., 2019). Furthermore, some cephalopod species may be difficult to manipulate without sedation or anesthesia (Oestmann et al., 1997). This is especially important when handling octopuses, since the strength, flexibility, and dexterity of their arms can easily interfere or difficult handling and/or procedures (Gleadall, 2013b).

*Octopus maya* (Voss and Solís Ramírez, 1966) is an endemic warm-water species of the Yucatan Peninsula, which inhabits shallow waters of the continental shelf at depths ranging from 0 to 50 m (Jereb et al., 2014). This species has a great social and economic importance for fisheries and is considered one of the most promising candidates for aquaculture diversification (for review, see Rosas et al., 2014). Studies in many areas, such as reproduction, embryonic development, nutrition, physiology, immunology, and climate change, have been carried out for *O. maya* (Domingues et al., 2007; Rosas et al., 2008; Avila-Poveda et al., 2009, 2016; Juárez et al., 2015, 2016; Linares et al., 2015; Caamal-Monsreal et al., 2016; Roumbedakis et al., 2018; López-Galindo et al., 2019; Pascual et al., 2019). However, to date, no information regarding anesthesia is available for this species.

This study explored, for the first time, the use of different substances (or cold sea water) to be used as potential anesthetic agents in *O. maya* juveniles. Specifically, we aimed to: (1) investigate the efficacy of a range of presumed anesthetic agents to induce a general anesthetic state in *O. maya* juveniles; (2) explore the metabolic consequences of acute exposure to the presumed general anesthetic agents; (3) analyze possible longer-term effects of repeated general anesthesia in the juvenile cephalopod *O. maya*; (4) define appropriate agent(s) for short-term handling and non-invasive procedures in *O. maya* juveniles.

## Materials and Methods

### Ethics Statement

In Mexico, cephalopods used for scientific purposes are not included in any animal welfare regulation. However, the experiments described here were previously submitted and approved at institutional level by the Animal Ethics Committee of the Faculty of Chemistry at Universidad Nacional Autónoma de México (UNAM, Sisal) (Permit Number: Oficio/FQ/CICUAL/099/15). The studies were conducted in accordance with the published Guidelines for the Care and Welfare of Cephalopods (Fiorito et al., 2015), which align with the principles of the European Directive (2010/63/EU) regulating animal research, including cephalopods, in the European Union (EU) (Smith et al., 2013). The recommendations of the ARRIVE Guidelines^1^ (Kilkenny et al., 2010) for reporting *in vivo* animal research were followed as closely as possible.

### Experimental Animals: Origin, Husbandry, and Housing

Juvenile *O. maya* were obtained from eggs of wild-caught adults captured by the traditional fishing method “gareteo” (small drifting boats with bamboo sticks placed at the bow and stern in which artisanal fishing lines containing crabs as baits are attached; Pech-Puch et al., 2016) off the coast of Sisal, Yucatan, Mexico. After capture, the adults were transported in tanks containing aerated sea water and kept at the laboratory of the UNAM, Sisal, according to best practices for this species as described by Rosas et al. (2014). Fertilized females were housed individually in 320-L rectangular dark tanks until spawning. The animals were fed twice a day with frozen crabs (*Callinectes* spp. weighing about 130 g; half crab in the morning and half in the afternoon). The tanks were cleaned daily to remove food leftovers and feces. Water quality parameters were monitored daily and maintained within the range recommended for *O. maya* (Rosas et al., 2014). Food was withdrawn if either the female octopus stopped feeding spontaneously or when spawning was observed.

After spawning, the eggs were artificially incubated for approximately 45 days, following the protocol established for this species (see details in Rosas et al., 2014). Hatchlings were cultured for around 2 months in 7.5 m^2^ (5.0 × 1.5 × 0.4 m) rectangular dark tanks at a density of 50–60 individuals m^–2^. Each tank contained at least one or two gastropod shells (*Melongena corona bispinosa* or *Strombus pugilis*, depending on the juvenile’s size) per animal to provide refuge. Animals were fed twice a day *ad libitum*, with a semi-dried crab-squid paste bound with gelatine (Rosas et al., 2008; Vidal et al., 2014). Every 20 days, the animals were weighed and separated according to size (+1 g of wet weight) to reduce competition and avoid cannibalism. During embryonic development and hatchlings rearing, temperature, salinity, pH, and dissolved oxygen (DO) concentration were maintained within the adequate ranges for this species, as previously described (Rosas et al., 2014). For the experiments, 2-month-old juveniles were housed individually as described below. The animals were fed, twice a day *ad libitum*, using the same diet as the hatchlings. During the experimental period, the plastic containers housing the juvenile octopuses (see details below) were cleaned daily to remove food leftovers and feces. Sea water quality parameters were recorded in a daily basis and maintained within the adequate ranges for this species, as follows: 24 ± 1°C; 35–38 PSU, pH >8, DO >5 mg L^–1^ and a 10:14 light/dark photoperiod regime with an intensity of 30 lux cm^2^.

### Investigation of Potential Anesthetic Agents

#### Study Design

A total of 98 juveniles (*n* = 7 animals/treatment), aged approximately 2 months, were weighed (wet weight mean ± SD: 1.67 ± 0.5 g) on a digital semi-analytical balance (OHAUS Scout SC2020) and randomly assigned to the experimental groups (see below; Table 1). The sex of the juveniles cannot be determined at this age. The animals were housed individually in square plastic containers (500 mL) inside a tank with recirculating sea water to acclimatize for 10-days. Each container had two apertures (∼5 × 7 cm) covered with plastic mesh (5 mm) on each side to allow the flow of water (Supplementary Figure S1); the meshes were cleaned daily. A gastropod shell (*Strombus pugilis*) was provided as den.

**TABLE 1:** Summary of the experimental groups: *Octopus maya* juveniles (*n* = 98; wet weight 1.67 ± 0.5 g) exposed or not to the substances (or cold sea water) to be used as potential anesthetic agents.

Experimental group	Concentration	Temperature
SW − H	–	25°C
SW + H	–	25°C
SW 11°C	–	11°C
SW 13°C	–	13°C
EtOH 0.5%	0.5%	25°C
EtOH 1.5%	1.5%	25°C
EtOH 3.0%	3.0%	25°C
MgCl_2_ 0.75%	0.75%	25°C
MgCl_2_ 1.5%	1.5%	25°C
MgCl_2_ 3.75	3.75%	25°C
Mix 1.5:0.75%	1.5% EtOH:0.75% MgCl_2_	25°C
Mix 0.75:1.13%	0.75% EtOH:1.13% MgCl_2_	25°C
Mix 2.25:0.37%	2.25% EtOH:0.37% MgCl_2_	25°C
Clove oil	0.15 mL L^–1^	25°C

*SW: sea water (25°C, unless if specified); SW − H: sea water, without handling; SW + H: sea water, with handling (see text for details). EtOH, ethanol; MgCl_2_, magnesium chloride; Mix, ethanol in combination with magnesium chloride.*

**FIGURE 1:**
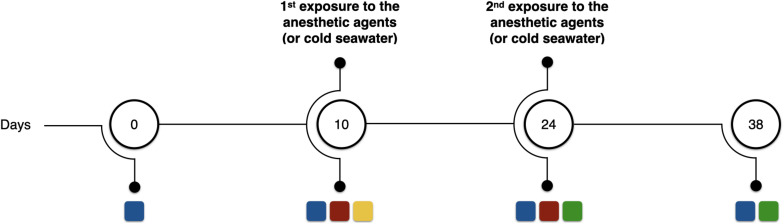
Schematic representation of the experimental timeline. Blue squares represent wet weight measurements; red squares assessment of criteria to anesthesia; yellow square represents oxygen consumption measurements before, during, and after exposure to the presumed anesthetic agents; and green squares represent assessment of mortality rates in relation to the precedent anesthesia. Numbers inside the circles represent the day of the experimental period in which the analyses were carried out.

After the acclimatization period, the oxygen consumption in sea water before, during and after exposure to the potential anesthetic agents was measured in a continuous flow respirometer (see details below). Subsequently, the octopuses were reared for another 14 days, and were then once again exposed to the potential anesthetic agents and the criteria for general anesthesia was analyzed. In the second exposure, each animal was exposed to the same substance and concentration as used during the first exposure (for oxygen consumption measurements purposes). Following, octopuses were reared for another 14 days. Surviving animals were used in subsequent studies in the laboratory following recovery. The experimental timeline is summarized in Figure 1, which also shows the key measurements made at each time point.

#### Oxygen Consumption Measurements

Oxygen consumption (VO_2_) was measured using a continuous flow respirometer comprised of a closed chamber connected to a recirculating sea water system (Rosas et al., 2008). Prior to the measurements, the juvenile octopuses were fasted for 24 h. Then, the animals were placed in 90 mL respirometric chambers with an approximate sea water flow rate of 100 mL min^–1^, in which they remained for 12 h before the experimental measurements. A *St. pugilis* shell was placed inside each chamber as a den for the octopuses. A chamber without any octopus but containing a shell was used as a control. Measurements of DO were recorded every 15 s for each chamber (input and output) with oxygen sensors attached to flow-cells connected by optical fiber to an Oxy 10 mini-amplifier (PreSens, Germany). The sensors were calibrated with seawater gassed with air to produce sea water with 100% DO and with a 5% sodium sulfate solution (0% DO). Sea water temperature in the chambers was maintained at 25°C, except during exposure to cold sea water (see Table 1 for details).

Data of oxygen consumption obtained 45 min before anesthesia were considered as a baseline of the treatments. Subsequently, the potential anesthetic agent or cold sea water was introduced into the chambers via the flow-through seawater system that filled the chambers. This procedure allowed a gradual exchange (around 1 min) of the water in the chambers for the potential anesthetic solution or cold sea water, without provoking an additional stress to animals. When the octopuses met the criteria for general anesthesia (see below) they were immediately removed from the respirometer, transferred to a container (exposed to air) for a standardized brief period of handling and weighing (180 s; protocol comparable to Gonçalves et al., 2012). While the animals were being handled, the respirometric chambers were emptied and refilled with fresh aerated sea water at 25°C and 35–38 PSU. After handling, the animals were returned to the respirometric chambers for recovery and the oxygen consumption measured for another 45 min. At the end of this period, food was offered to the octopuses and the presence of an attack response within 300 s was used as an indicator of recovery as described by Gonçalves et al. (2012) for *Sepia officinalis*. The animals were then returned to their individual home plastic containers.

#### Criteria for General Anesthesia

Criteria for general anesthesia in cephalopods has been the subject of several original publications (Andrews and Tansey, 1981; Messenger et al., 1985; Gonçalves et al., 2012; Polese et al., 2014; Butler-Struben et al., 2018) and reviews (Andrews et al., 2013; Gleadall, 2013b; Fiorito et al., 2015). In this article, investigating potential agents for the first time in *O. maya* we used the following criteria: (i) a decrease in chromatophore tone (paling) of the skin of the mantle, head and arms; (ii) a loss of muscle tone manifest as flaccidity of the arms and mantle; (iii) reduced or absent sucker adhesiveness indicated by inability to adhere to the walls of the chamber or by touching; (iv) loss of normal posture and immobility; (v) cessation of breathing indicated by absence of obvious contraction of the mantle; (vi) absence of a response to a noxious mechanical stimulus (pinch of the skin of the arms or the orbit). To assess general anesthesia, the octopuses were transferred from their home plastic containers to a circular, white plastic container (250 mL). The time to induce general anesthesia is defined as the time at which all the above criteria were met after introduction of the animal in the potential anesthetic solution. However, as one of the aims of this study was to identify agents which could be used to induce anesthesia relatively quickly for routine handling and non- or minimally invasive procedures and from which the animals would recover quickly we set a maximum time of 600 s for an agent to fulfill the criteria (Gonçalves et al., 2012). Time to recovery is the time after transfer to aerated sea water to when the animals were breathing, had regained a normal posture and colouration as assessed by visual inspection. A 600 s cut off was used for measurement of recovery time. In the first part of the experiment, during which animals were exposed to the potential anesthetic solutions in the respirometer chambers, it was only possible to visually assess criteria (i) to (v) as described above. In the subsequent exposure, carried out in an open chamber, all six criteria were assessed. In both studies, the same handling and attack response protocols were used, however, after the first exposure, the attack response was assessed after the full period of oxygen consumption measurements during recovery (45 min) while in the second exposure, the attack response was assessed immediately after recovery. The attack response was recorded only as positive (+) or negative (−). During all the experiments, data were assessed always by the same two people and in such way that one of them made the observations and the other made the records.

#### Growth and Mortality

Possible longer-term effects of repeated exposure to the anesthetic agents were determined by measuring growth and mortality. The weight of the animals was determined at the beginning and at the end of the experimental protocol, as well as at each anesthesia event (during handling) (Figure 1). Daily growth coefficient (DGC) expressed as %BW d^–1^, where BW is body weight (wet weight) in g. DGC was calculated for each octopus taking into account the experimental periods in which the animals were anesthetized and/or manipulated (day 0 to 10, day 10 to 24, and day 24 to 38); total growth rate was calculated considering all the experimental period (from day 0 to 38). DGC was calculated using the equation: DGC (%BW d^–1^) = [(Ln BW_2_ − Ln BW_1_)/*t*] × 100, where BW_2_ and BW_1_ are the final and initial wet weights of the juveniles, respectively, Ln the natural logarithm and *t* the number of days of the experimental period. Mortality was calculated as the number of deaths in the 14 days following each anesthetic exposure.

### Experimental Groups

Exposure to cold sea water (SW + temperature), ethanol (EtOH), magnesium chloride (MgCl_2_), combinations of ethanol with magnesium chloride (Mix), and clove oil were selected to be tested for their potential as general anesthetic agents (for details, see Table 1) for *O. maya* juveniles. Anesthetic agent concentrations were selected based on the literature (Andrews and Tansey, 1981; Messenger et al., 1985; Mooney et al., 2010; Gonçalves et al., 2012; Gleadall, 2013b; Fiorito et al., 2015). The temperatures of cold sea water were based on the Critical Thermal Minima (CTMin) established for *O. maya* juveniles by Noyola et al. (2013a) at which the metabolic rate is nearest to zero (i.e., 11.6°C). Exposure to sea water at 11 and 13°C was achieved by decreasing the temperature with bottles of frozen sea water. The solutions containing the anesthetic agents were prepared just before use. Ethanol (Merck, Mexico) concentrations (0.5–3.0%) were obtained by direct dilution in sea water. Solutions containing magnesium chloride (Hexahydrate; Sigma Aldrich, Mexico) (0.75–3.75%) were obtained adjusting distilled and sea water volumes, in order to maintain the salinity similar to the sea water of the acclimation tank. For clove oil (Sigma Aldrich, Mexico), a stock solution was dissolved in 96% ethanol at a ratio of 1:10 and then diluted in sea water to obtain the final concentration (0.15 mL L^–1^). Two experimental groups not exposed to any anesthetic agent, without (SW − H) or with handling (SW + H), were also evaluated (Table 1).

#### Statistical Analysis

All data were tested for normality distribution. Chi-squared test was used to compare if anesthetic agents induce complete anesthesia and affect the incidence of attack response after recovery. Data on wet weight were transformed on Ln and analyzed by linear regression. ANCOVA analysis was applied to known differences between changes in time of wet weight of animals manipulated along the experimental period. The result of this analysis showed that the changes in wet weight of octopus anesthetized with clove oil were statistically different from octopus from other treatments. For this reason, a new ANCOVA analysis was developed comparing data of wet weight changes of animals exposed to clove oil and the remaining treatments, including the treatments were animals were not exposed to any anesthetic agent. Regression analysis and ANCOVA tests were done using Prism software (6.0). To evaluate if the anesthetic agent and handling affected the growth rate of the animals (%BW d^–1^), a one-way ANOVA was applied to the data at the end of the experiment. Additionally, one-way ANOVA followed by Newman–Keuls was used to test the effect of the anesthetic agents on induction and recovery times of animals anesthetized and manipulated. Oxygen consumption measurements were analyzed considering three different stages: baseline (before anesthesia) (all 14 treatments: 12 exposed to the anesthetic agents or cold sea water and two not exposed); anesthesia (11 treatments, excluding the treatments in which animals were not exposed to any anesthetic agent and the treatment in which the animals were exposed to clove oil, since this treatment produced non-detectable values of oxygen consumption, considered as zero) and recovery (after anesthesia and/or handling) (all 14 treatments). The results for the baseline were analyzed using a one-way ANOVA. The effects of each treatment and the interaction with the time of exposure were considered while analyzing the data obtained from the animals during and after anesthesia. Each group of data, anesthesia, and recovery were analyzed separately with a two-way ANOVA, followed by the Tukey Honesty test to determine significant differences among the experimental groups. In ANOVA tests, homogeneity of variances was evaluated using the Cochran test. A histogram was used to test the normality of the data. Differences were considered statistically significant at the *p* < 0.05. For all ANOVA tests we used STATISTICA software.

## Results

### Oxygen Consumption Measurements

**FIGURE 2:**
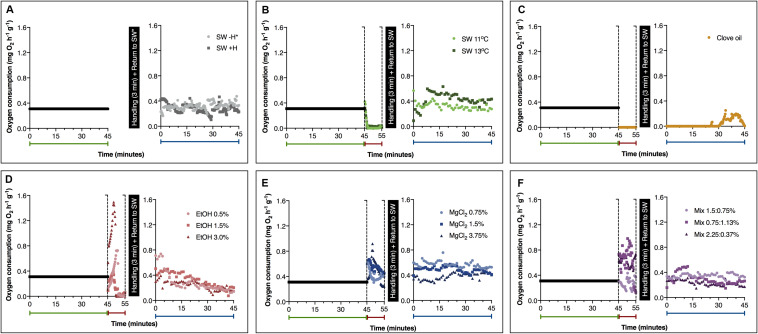
Oxygen consumption of *Octopus maya* juveniles in sea water and before, during and after the exposure to different substances (or cold sea water). Green, red and blue labels represent oxygen consumption before (baseline), during and after anesthesia and/or handling (recovery), respectively. **(A)** Animals not exposed to any anesthetic agent; Animals exposed to **(B)** cold sea water; **(C)** clove oil; **(D)** ethanol; **(E)** magnesium chloride; and **(F)** ethanol in combination with magnesium chloride. SW: sea water (25°C, unless if specified); SW – H: sea water, without handling; SW + H: sea water, with handling (see text for details). EtOH, ethanol; MgCl_2_, magnesium chloride; Mix, ethanol in combination with magnesium chloride (concentrations are provided in this order). *SW – H measurements were performed without any interruption. Data obtained from the first exposure to anesthesia. Note: As the baseline oxygen consumption was similar in the experimental groups (*p* > 0.05), we used the calculated mean value as a visual reference of the baseline oxygen consumption of animals (see section “Results” for details).

Mean values on oxygen consumption obtained from animals exposed to the different substances (or cold sea water) before, during (first 600 s) and after anesthesia and/or handling are presented in Figure 2. Baseline oxygen consumption (i.e., before exposure to the presumed anesthetic agents) was similar in the experimental groups (*p* = 0.052; *F* = 93.28). For this reason, a mean value was calculated (0.31 ± 0.09 mg O_2_ h^–1^ g^–1^). During exposure to the presumed anesthetic agents, the results on oxygen consumption showed that the metabolic rate was affected by treatment and time of exposure (Table 2). In octopuses exposed to both temperatures of cold sea water (11 and 13°C), a significant decrease on oxygen consumption was recorded after 120 s of exposure, producing non detectable values (considered here as zero) of metabolic rate (*p* < 0.00001) (Figure 2B). Juvenile octopuses exposed to different concentrations of EtOH also showed a significant reduction on oxygen consumption to non-detectable values (Figure 2D). These values were reached earlier on animals exposed to EtOH 3.0% (after 285 s of exposure) than in animals exposed to EtOH 0.5 and 1.5% (after about 360 s of exposure) (*p* < 0.014). However, it should be noted that animals exposed to EtOH 3.0% showed an increase on oxygen consumption after 180–240 s of exposure, reaching the highest values registered among all treatments (Figure 2D). The mean value calculated for this period of exposition to EtOH 3.0% was 1.46 ± 0.4 mg O_2_ h^–1^ g^–1^, 4.7 times higher than the mean value calculated for the baseline. A part from hyperventilation, represented by an initial increase in oxygen consumption of the animals exposed to EtOH concentrations during the first 300 s of exposure, no apparent stressful reactions were observed in these animals. In general, oxygen consumption of the animals exposed to all concentrations of MgCl_2_ remained practically constant during the 600 s of exposure. A significant increase on oxygen consumption of octopuses exposed to MgCl_2_ 3.75% after 180–210 s of exposure was observed, with values reaching 0.84 ± 0.3 mg O_2_ h^–1^ g^–1^ (*p* < 0.01). No significant differences were observed in the other values registered (*p* > 0.05; Figure 2E). In octopuses exposed to Mix 1.5:0.75%, oxygen consumption in the first 260 s of exposure was lower (0.23 ± 0.05 mg O_2_ h^–1^ g^–1^) than in the animals exposed to Mix 0.75:1.13% and 2.25–0.37% (mean values of 0.66 ± 0.16 mg O_2_ h^–1^ g^–1^) (*p* < 0.0012; Figure 2F). Likewise, an increase on the oxygen consumption of the animals exposed to Mix 1.5:0.75% was registered between 360 and 450 s of exposure, with mean values of 0.82 ± 0.17 mg O_2_ h^–1^ g^–1^ (*p* < 0.023), followed by a quick reduction reaching 0.15 ± 0.02 mg O_2_ h^–1^ g^–1^ after 540–600 s of exposure (*p* < 0.0134; Figure 2F). Exposure to clove oil cause a reduction in the oxygen consumption of the animals during anesthesia, however, a full recovery with values of oxygen consumption comparable to the baseline was missing at the end of 45 min after exposure (Figure 2C).

**TABLE 2:** Results of two-way ANOVA on oxygen consumption of *Octopus maya* juveniles exposed to the different anesthetic agents.

	SS	DF	MS	*F*	*p*
Anesthetic agent	131.6	9	14.62	320.6	0.00*
Time	31.5	39	0.81	17.7	0.00*
Anesthetic agent vs time	103.1	351	0.29	6.4	0.00*

This analysis was made considering the data obtained from the 11 experimental groups, excluding the groups in which the animals were not exposed to any anesthetic and the data obtained from animals anesthetized with clove oil, since this treatment produced only non-detectable values of oxygen consumption (considered as zero). *Statistical differences at *p* < 0.0001 level.

After manipulation and during recovery, in animals not exposed to any anesthetic agent, oxygen consumption was similar to the values registered during baseline (Figure 2A). In *O. maya* juveniles exposed to SW 11°C, the oxygen consumption during recovery was comparable to the baseline, with a mean value of 0.32 ± 0.10 mg O_2_ h^–1^ g^–1^ (Figure 2B). Similarly, oxygen consumption of the animals exposed to SW 13°C was also comparable to baseline oxygen consumption in the first 10 min of recovery. Although, a small increase of oxygen consumption was detected after this period in these animals when compared to octopuses exposed to SW 11°C, at the end of the recovery period, both groups presented values comparable to baseline oxygen consumption (Figure 2B). Values of oxygen consumption comparable to the baseline were also observed in juvenile octopuses exposed to EtOH, MgCl_2_, and Mix (Figures 2D–F), except by animals exposed to MgCl_2_ 0.75%, which showed, at the end of the recovery period, oxygen consumption significantly higher than the ones registered during the baseline (Figure 2E; *p* < 0.01).

### Criteria for General Anesthesia

**FIGURE 3:**
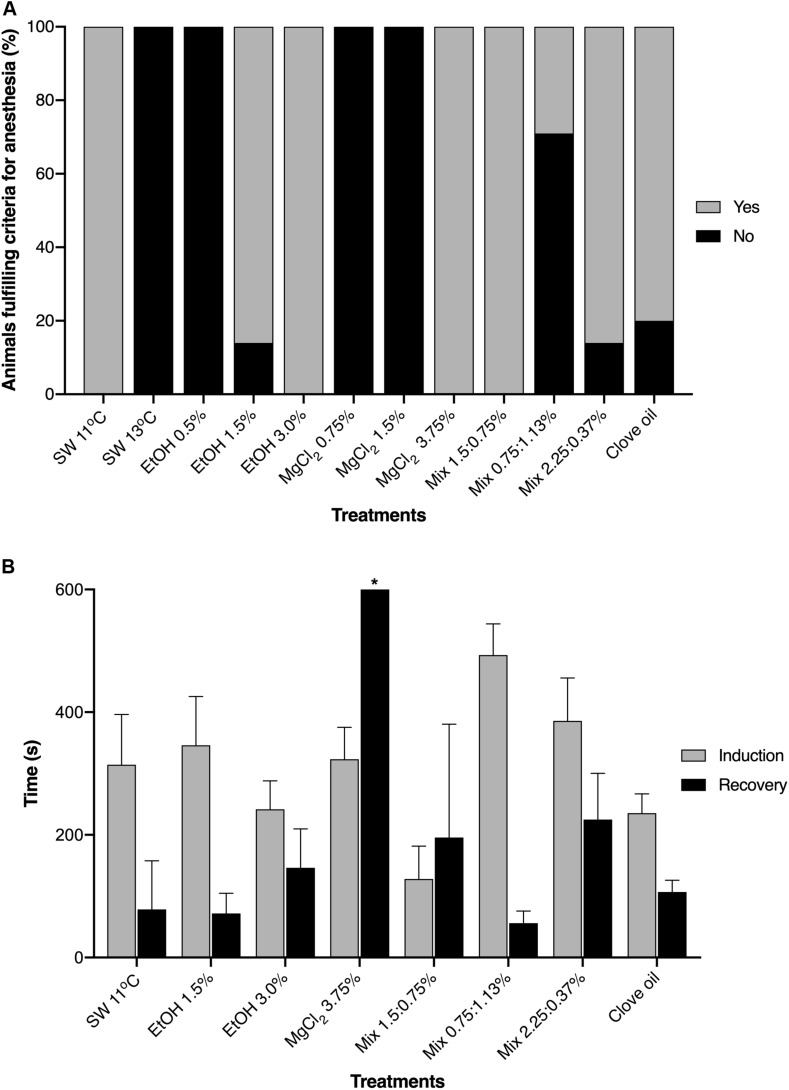
**(A)** Percentage of *Octopus maya* juveniles that fulfilled the criteria (see section “Materials and Methods”) for complete induction to general anesthesia when exposed to different substances (or cold sea water). **(B)** Times of induction and recovery from anesthesia. SW, sea water; EtOH, ethanol; MgCl_2_, magnesium chloride; Mix, ethanol in combination with magnesium chloride (concentrations are provided in this order). Data obtained from the second exposure to anesthesia. *Animals exposed to MgCl_2_ 3.75% did not fulfill the criteria for recovery from anesthesia in the pre-established time of 600 s (which does not mean that they did not recovery from anesthesia).

Except for clove oil, all other anesthetic agents induced (in a given concentration) general anesthesia in the juvenile *O. maya*. All animals exposed to sea water at 11°C, EtOH 3.0%, MgCl_2_ 3.75% and Mix 1.5:0.75% fulfilled all the criteria for general anesthesia in the pre-established time (600 s). Ethanol 1.5% and Mix 2.25:0.37% induced general anesthesia in 86% of the animals, whereas only 26% exposed to Mix 0.75:1.13% fulfilled all the criteria. In contrast, all animals exposed to sea water at 13°C, EtOH 0.5%, MgCl_2_ 0.75 and 1.5% and clove oil did not fulfill the criteria for induction to anesthesia (Figure 3A). Although the juveniles exposed to clove oil met some of the criteria (except for criteria ii and iii), typical adverse response pattern (TARP) as described by Gleadall (2013a) were observed in all animals. Reactions included fast disoriented swimming behavior and erratic swimming movements, attempts to escape and jetting against the container’s wall, violent contraction of the mantle, sometimes causing inability to breath (closing of the funnel), rapid color changes, exaggerated raising papillae, abnormal lageniform appearance of the mantle (see Gleadall, 2013b for details) and inking. One animal was not able to completely release the ink due to mantle rigidity and closure.

Induction and recovery times (see section “Materials and Methods” for definitions and protocol) in the animals that fulfilled the criteria for general anesthesia are presented in Figure 3B. Induction times were similar between the treatments with exception of animals exposed to EtOH 3.0% which had lower induction times (*p* < 0.05) when compared to animals exposed to Mix 2.25:0.37%. On the other hand, animals exposed to sea water at 11°C, EtOH 1.5% and Mix 2.25:0.37% recovery was slower (*p* < 0.05) than animals exposed to Mix 0.75:1.13%.

**FIGURE 4:**
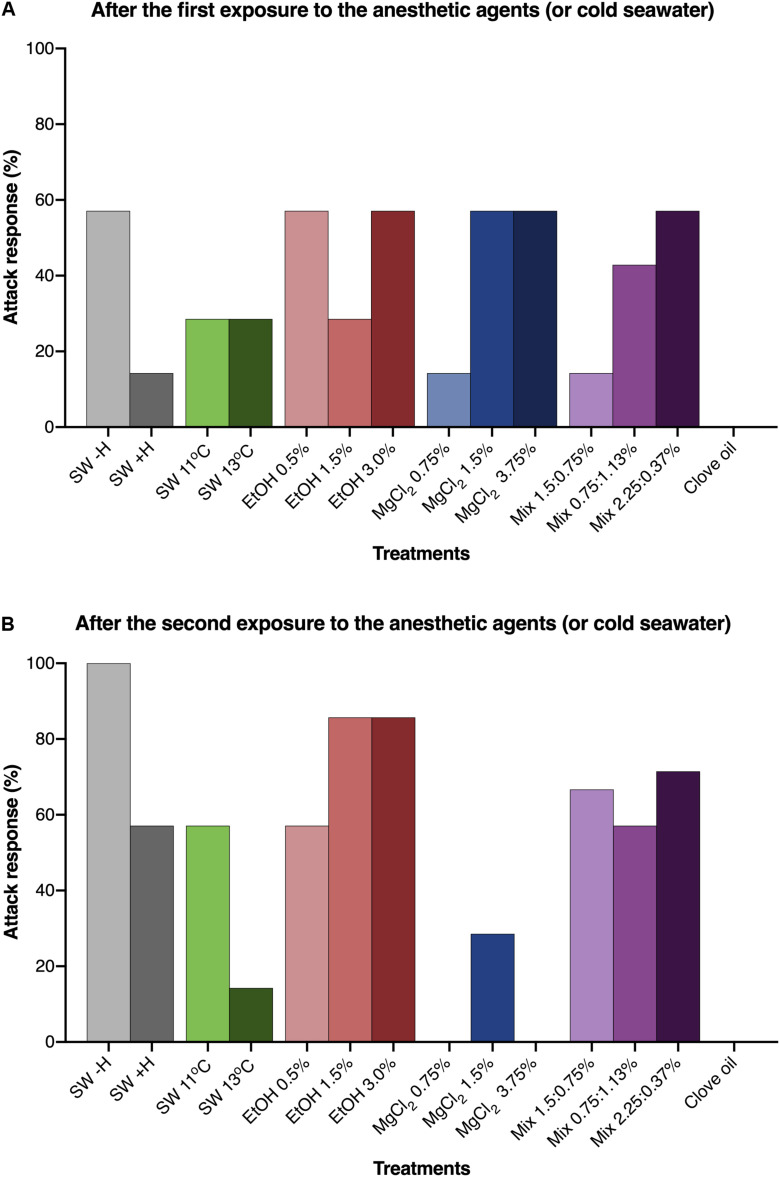
Percentage of *Octopus maya* juveniles that presented attack response when food was offered after the first **(A)** and the second **(B)** exposure to the presumed anesthetic agents (or cold sea water) and in the experimental groups that were not exposed to any anesthetic agent (SW – H; SW + H). SW: sea water (25°C, unless if specified); SW – H: sea water, without handling; SW + H: sea water, with handling (see text for details). EtOH, ethanol; MgCl_2_, magnesium chloride; Mix, ethanol in combination with magnesium chloride (concentrations are provided in this order). Chi^2^ analysis were made to test if the attack response were affected by the type of presumed anesthetic after the first **(A)** and the second **(B)** exposure. For **(A)**: Calculated Chi^2^ = 38.1; Table Chi^2^ = 31.3; GF = 14; *p* < 0.005, in this analysis two experimental groups in which the animals were not exposed to the anesthetic agents were added. For **(B)**: Calculated Chi^2^ = 63.2; Table Chi^2^ = 19.67; GF = 11; *p* < 0.001. Both testes indicate that the type of presumed anesthetic affected significantly the attack response of *O. maya* juveniles in two testes **(A,B)**.

The use of anesthetic agents and/or handling affected the attack response of *O. maya* juveniles (*p* < 0.001). Animals that were not exposed to anesthesia or handling (SW − H) promptly responded to the presence of food, whereas in animals submitted only to handling (SW + H), a reduced incidence of attack response was observed. After exposure to EtOH 1.5 and 3.0%, more than 80% of the animals showed predatory behavior. In animals exposed to SW at 11°C, EtOH 0.5% and all concentrations of the mixed solutions, incidence of attack responses varied between 57 and 71%. On the other hand, incidence of attack response was reduced or inhibited in animals exposed to all concentrations of MgCl_2_, SW 13°C and clove oil (Figure 4).

### Growth and Mortality

**FIGURE 5:**
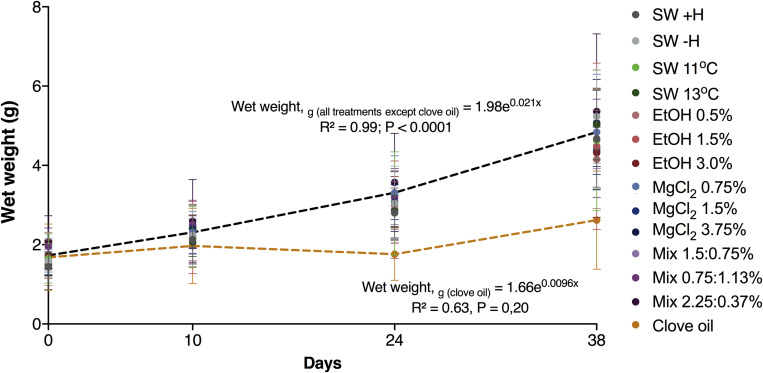
Mean wet weight (±SD) of *Octopus maya* juveniles exposed or not to the anesthetic agents. All treatments except clove oil (continuous line): Wet weight (g) = 1.60e^0.28 (^*^T^*^, days)^; clove oil (dashed line): wet weight (g) = 1.53e^0.0089 (^*^T^*^, days)^. ANCOVA analysis showed differences in slopes at *p* < 0.0006 levels between clove oil and the rest of the treatments (see Supplementary Table S2).

**FIGURE 6:**
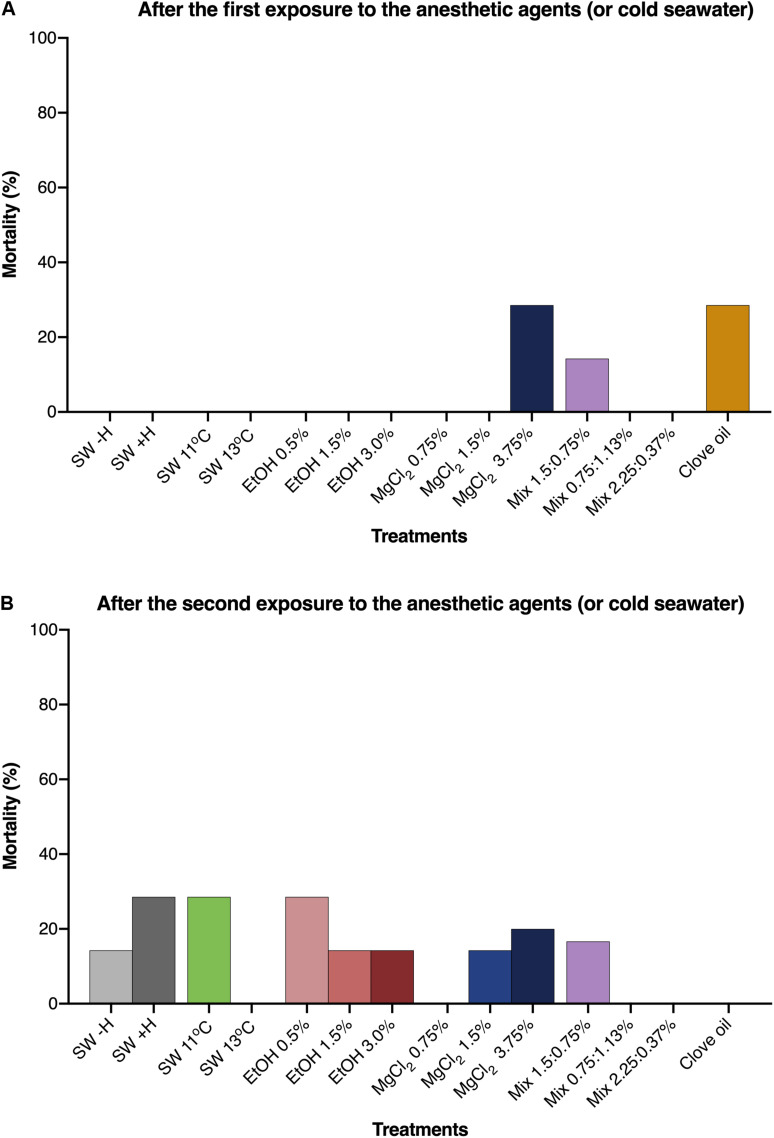
Mortality rates of *Octopus maya* juveniles after the first **(A)** and the second **(B)** exposure to the anesthetic agents (or cold sea water) and in the experimental groups that were not exposed to any anesthetic agent (SW – H; SW + H). SW: sea water (25°C, unless if specified); SW – H: sea water, without handling; SW + H: sea water, with handling (see text for details). EtOH, ethanol; MgCl_2_, magnesium chloride; Mix, ethanol in combination with magnesium chloride (concentrations are provided in this order).

Except for the animals exposed to clove oil, the growth of *O. maya* juveniles was similar in all other groups [wet weight (g) = 1.98e^0.021 (^*^T^*^, days)^; Figure 5]. The animals exposed to clove oil had a reduced growth rate compared to all other experimental groups after the first and second exposure [*p* < 0.0006; wet weight (g) = 1.66e^0.009 (T, days)^; Figure 5, Supplementary Tables S1, S2, and Supplementary Figure S2]. Mortality of the juveniles after the first and the second exposure to the anesthetic agents are presented in Figures 6A,B, respectively. After the first exposure, mortality was observed only in animals exposed to MgCl_2_ 3.75%, Mix 0.75:1.5%, and clove oil. Nevertheless, after the second exposure, natural mortality was observed in nine of the 14 experimental groups, including in the groups where animals were not exposed to anesthesia (i.e., SW − H and SW + H).

## Discussion

Here, we provide, for the first time, original data regarding anesthesia in the warm-water species *O. maya*, including the physiological effects of exposure to anesthetics on octopuses’ oxygen consumption. We also include additional information on possible longer-term effects of repeated anesthesia on the growth and mortality of juvenile octopuses. As one of our goals was to define an agent which induces anesthesia in a short period (less than 600 s) to perform quick handling (>180 s), we focused the discussion mainly in the substances considered, according to our requisites, adequate for these purposes. For this reason, we will not discuss in-depth the data obtained from animals exposed to clove oil, since it is clearly not suitable to anesthetize this species. Although our experiments were carried out with *O. maya* juveniles, TARP was also observed in adult *O. maya* (unpublished data), thus, we strongly believe that clove oil does not produce effective anesthesia in this species.

### Considerations Regarding Anesthesia and Anesthetic Agents in Cephalopods

Immersion baths in the anesthetic solution are the most commonly method utilized to anesthetize cephalopods. A range of substances, such as urethane, ethanol, tricaine methanesulfonate (MS-222), benzocaine, clove oil, menthol, magnesium chloride, and sulfate, as well as cold sea water, have been tested as anesthetic agents for cephalopods (see review in Gleadall, 2013b; Fiorito et al., 2015). However, the use of some of these substances were discontinued due to negative and stressful reactions caused to the animals during exposure, low effectiveness of anesthesia, or toxicity to humans. Despite the potential of use of volatile (e.g., isoflourane and sevoflourane) and injectable (e.g., ketamine and propofol) anesthetic agents, commonly utilized in vertebrates, studies with these agents in cephalopods are rare or inexistent (Polese et al., 2014).

The most commonly used substances and considered, to date, the most effective in anesthetizing cephalopods are the solutions containing ethanol and magnesium chloride, used separately or in combination (Gleadall, 2013b; Fiorito et al., 2015; Butler-Struben et al., 2018). Both substances are of relatively low cost and ease access, manipulation and application. Although ethanol and magnesium chloride are generally not accepted to anesthetize animals from other taxa, satisfactory results have been obtained in cephalopods.

Ethanol in concentrations between 1.0 and 3.0% have been successfully used to anesthetize different species of cuttlefishes, squids and octopuses (for review, see Gleadall, 2013b; Fiorito et al., 2015). However, some authors (Froesch and Marthy, 1975; Andrews and Tansey, 1981) described adverse reactions of the animals after immersion in the anesthetic solution, such as escape attempts and inking. In addition, incomplete induction may occur at low temperatures (Gleadall, 2013b), probably due to a reduction of the narcotizing effect of ethanol (Moore et al., 1964). For this reason, solutions containing ethanol might be ineffective to anesthetise cold-water species.

Magnesium chloride is probably the oldest substance used for anesthetizing invertebrates (Ross and Ross, 2008). Successful induction to anesthesia using different formulations of magnesium chloride (i.e., diluted directly in sea water or in a combination of sea water and distilled water) have been achieved for cephalopod species of different sexes, ages, and sizes (Messenger et al., 1985; Gore et al., 2005; Gonçalves et al., 2012; Gleadall, 2013b; Pugliese et al., 2016; Butler-Struben et al., 2018). Likewise, magnesium chloride had been effectively used for long-duration anesthesia (Mooney et al., 2010). Nonetheless, similarly to ethanol, stressful reactions and inking were eventually noted (Garcia-Franco, 1992). Controversial opinions whether magnesium chloride produces adequate general anesthesia or simply act as neuromuscular blocking agent are discussed in the literature (Andrews et al., 2013; Polese et al., 2014; Fiorito et al., 2015; Butler-Struben et al., 2018; Winlow et al., 2018), however, no consensus is completely achieved to date.

Cold sea water (commonly also referred as hypothermia) as anesthetic agent has the advantage of avoiding the use of chemicals (and potential associated negative effects) (Gleadall, 2013b) and, despite the popularity of use in the past, its anesthetic properties are questionable (Andrews and Tansey, 1981; Gleadall, 2013b; Fiorito et al., 2015; Winlow et al., 2018). Muscle relaxation is usually reported to lack in animals exposed to cold sea water (e.g., Andrews and Tansey, 1981). Immersion in cold sea water may be effective for immobilization rather than inducing general anesthesia (Gleadall, 2013b) and thus, its use in potentially invasive procedures should not be considered.

Clove oil has been successfully used to anesthetize many species of fish (Taylor and Roberts, 1999; Javahery et al., 2012; Priborsky and Velisek, 2018) and aquatic invertebrates (Araujo et al., 1995; Bilbao et al., 2010). Although the potential use of clove oil was speculated for humanely killing cephalopods (Andrews et al., 2013; Fiorito et al., 2015) effectiveness of anesthesia using this agent in some species is uncertain. Satisfactory results were reported only for *O. minor* (Seol et al., 2007). For other species (e.g., *O. vulgaris* – including different life stages, *Dorytheutis pealeii*, *Se. officinalis*), similarly to our results for *O. maya*, no anesthetic effect and/or highly stressful reactions were observed (Mooney et al., 2010; Estefanell et al., 2011; Gonçalves et al., 2012; Escánez et al., 2018).

### Effects of the Anesthetics on Animals’ Oxygen Consumption

As active animals, oxygen consumption rates of cephalopods are directly related to their activity (Segawa and Hanlon, 1988). For instance, the oxygen consumption measured during active swimming is higher than in resting cephalopods: these values can increase around 2.4 times in *O. vulgaris* (Wells et al., 1983b) up to 2.6–3.4 times in *Loligo opalescens* (O’Dor, 1982; Webber and O’Dor, 1985) and five times in *Illex ilecebrosus* (Hoeger et al., 1987). Similarly, oxygen consumption during and after feeding also increase when compared to fasted cephalopods (Hirtle et al., 1981; Wells et al., 1983a; Rosas et al., 2008).

In the present study, the mean value calculated for baseline oxygen consumption obtained from *O. maya* juveniles (i.e., 0.31 ± 0.09 mg O_2_ h^–1^ g^–1^) was similar to the values previously registered in fasted juveniles of the same species (0.31 ± 0.06 mg O2 h−1 g^–1^, Rosas et al., 2008; 0.31 ± 0.04 mg O_2_ h^–1^ g^–1^ at 22°C, Noyola et al., 2013b). After immersion in the anesthetic solution, hyperventilation is frequently reported in cephalopods (e.g., Andrews and Tansey, 1981; Messenger et al., 1985; Gonçalves et al., 2012) and, consequently, an increase in oxygen consumption is expected. Increased ventilation frequency can be considered an indicator of physiological stress (Fiorito et al., 2015). Thus, this should be reduced to a minimum (Gleadall, 2013a) and be followed by a significant reduction in ventilation frequency (and, consequently, in oxygen consumption), expected to occur as a result of an adequate anesthesia induction and lower activity levels of the animal.

In fact, our results indicate that animals exposed to different concentrations of EtOH, Mix 0.75:1.13%, and MgCl_2_ 3.75% showed an increase in oxygen consumption during the beginning of the exposure. Nonetheless, in general, the values registered during anesthetic exposure are comparable to the ones obtained for *O. maya* juveniles during feeding (up to 3.4 times higher than in fasted animals, depending on the diet) (Rosas et al., 2008). Although oxygen consumption obtained from the juveniles exposed to EtOH 3.0% was higher (∼4.7 times than baseline values), this lasted less than 300 s and was followed by a significant decrease in these values. Interestingly, in animals exposed to the mixed solutions (EtOH + MgCl_2_) this effect was much lower or absent when compared to the animals exposed to EtOH alone. This is probably due to the combined effects of both substances. The combination of substances to obtain general anesthesia, each contributing to the overall effect, is a common practice in laboratory animals (Flecknell, 2015) but still need to be better explored in cephalopods.

Exposure to cold sea water caused a direct reduction in oxygen consumption of the juvenile octopuses, as expected, since lower temperatures decreases animal’s metabolism. When considering the use of cold sea water to “tranquilize” or immobilize cephalopods, CTMin and time of exposure should be considered to avoid excessive stress. The exposure of animals to temperatures below the CTMin may induce disorganized locomotory responses, signs of stress and atypical behaviors, ultimately leading to death (Noyola et al., 2013a). Nonetheless, CTMin is not yet known for the great majority of the commonly studied species. In addition, to avoid a possible cold-shock stress, gradual cooling of sea water might be more appropriate.

The relationship between oxygen consumption and the exact moment at which the animal fulfills the criteria for anesthesia, which includes immobility and marked reduction or absence of respiration, should be further investigated. During recovery, almost all experimental groups presented values of oxygen consumption comparable to baseline values. There was no evidence for an overshoot in oxygen consumption after a period with reduced or no ventilation.

### Criteria for General Anesthesia and Requisites for Selecting Anesthetic Agents

Anesthetic agents and concentrations are frequently used indiscriminately of the species and following protocols already developed for the most commonly studied or cultured species (Zahl et al., 2012). However, the anesthetic agent and the protocol used for one species may not be appropriate for others or even for a diverse life stage of the same species. This is similarly important when defining criteria for anesthesia. For instance, Escánez et al. (2018) considered *O. vulgaris* paralarvae anesthetized when they “lost the ability to swim and remained perfectly still on the bottom,” which differs from the criteria commonly used for adults of the same species (Andrews and Tansey, 1981; Estefanell et al., 2011; Butler-Struben et al., 2018).

In the present study, we tried to be the most precise as possible when defining the criteria for assessing general anesthesia in order to facilitate the standardization of a protocol to be used for *O. maya* juveniles. The criteria defined here represent behavioral indicators that accomplish, in general lines, the definition of anesthesia. Although we do not accessed consciousness, a recent study has demonstrated that magnesium chloride and ethanol are considered “genuine anesthetics” for cephalopods (Butler-Struben et al., 2018).

One of the criteria considered to evaluate general anesthesia in cephalopods is the decrease and, eventually, cessation of ventilation (Gleadall, 2013b; Fiorito et al., 2015). Cessation of ventilation would be an unacceptable criterion for mammals. Therefore, this might be questionable for cephalopods as well. Although cutaneous oxygen uptake has been demonstrated in cephalopods (Wells and Wells, 1983; Madan and Wells, 1996) its contribution for oxygen acquisition only recently has been systematically explored (Birk et al., 2018). In addition, the effects on brain function and/or possible tissue damages after hypoxia are not well understood.

For our purposes, we define that a given anesthetic agent and concentration should produce quick induction and recovery (600 s), with side-effects and stressful reactions reduced to a minimum, and allow animals’ handling during 180 s. Following these definitions, except for clove oil, all the anesthetic agents tested induced (in a given concentration) general anesthesia in *O. maya* juveniles. All octopuses exposed to SW 11°C, EtOH 3.0%, and Mix 1.5:0.75% fulfilled the criteria defined for general anesthesia. Animals exposed to MgCl_2_ 3.75%, despite fulfilling the criteria for general anesthesia, presented recovery times exceeding 600 s.

Another relevant factor to be considered for anesthetic selection, are the induction and recovery times. Anesthesia should have rapid action, with stress reactions and hyperactivity, reduced to a minimum; similarly, recovery should not last long and none or minimum side-effects are desirable (Winlow et al., 2018). For fishes, recommended times of induction and recovery should be preferably not longer than 3 and 5 min, respectively (Ross and Ross, 2008). In the present study, times of induction were, in general, similar among the substances that induce complete anesthesia according to our criteria and varied from around 180 (EtOH 3.0%) to 480 s (Mix 0.75:1.13%). Recovery times were shorter, varying from around 60 s (SW 11°C) and 240 s (Mix 2.25:0.37%). Animals exposed to MgCl_2_ 3.75% did not recovery in 600 s, which does not mean that they did not recovery from anesthesia, but simply that they did not meet the requisites established for this study.

### Short- and Long-Term Effects of Anesthesia

The exposure to an anesthetic can result in important direct consequences in the physiology and behavior of the animals and eventually impair food intake, growth and other important aspects in the long-term (Ross and Ross, 2008). Our results show significant changes in attack response to food in animals exposed to the different anesthetic agents. The attack response in animals exposed to all concentrations of MgCl_2_, SW 13°C and clove oil, differently from the other experimental groups, was inhibited after the second exposure. This inhibition could be an adverse effect of repeated exposure to these agents. After the second anesthesia, animals exposed to EtOH 1.5 and 3.0% showed higher attack response when compared to animals exposed to other anesthetic agents and animals that were just handled (SW + H). Although different species may have distinct responses, similar results were observed by Gonçalves et al. (2012) in cuttlefishes exposed to EtOH and clove oil using similar concentrations. On the other hand, different responses in cuttlefishes exposed to MgCl_2_ were observed. It should be noted that these results might be influenced by species, size of animals and the applied methodology (including differences in concentration of some anesthetics).

To the best of our knowledge, long-term effects of anesthesia have never been explored in cephalopods. In the present study, clove oil was the only substance that affect *O. maya* juveniles’ growth, which reinforces that this agent is not suitable to anesthetise this species. A high number of deaths in the end of the experiment (four out of seven) was observed in animals exposed to MgCl_2_ 3.75%. Pugliese et al. (2016) observed that the worst cardiac performance of isolated hearts of *O. vulgaris* previously exposed to anesthetics was obtained with a concentration similar to the one used in the present study (i.e., MgCl_2_ 3.5% dissolved in sea- and distilled-water; 1:1). These findings could possibly explain the high mortality rate in animals exposed to MgCl_2_ 3.75% observed in our study. Nonetheless, we are aware that the data obtained for mortality should be analyzed with caution, since natural mortality was also observed in other experimental groups, including the ones where animals were not exposed to anesthesia.

## Conclusion

In conclusion, our study provides insights into the metabolic and behavioral responses of juvenile octopuses to anesthesia. Our results showed that during short-term handling (not longer than 180 s) of *O. maya* juveniles that do not cause pain, distress, suffering or lasting harm to the animals, the use of anesthetic agents can be eventually suppressed. If the handling will be carried out easily in an anesthetized/tranquilized animal, we suggest the use of EtOH 3.0% or cold sea water 11°C. These agents presented the most appropriate results according to our criteria: satisfactory metabolic responses; absence or reduced stressful reactions during induction and recovery from anesthesia; induction and recovery times within the maximum pre-specified time range (600 s); adequate growth and low mortality rates. The data presented here might serve as a reference for other cephalopod species, specially, juvenile warm-water octopuses. Ultimately, the protocol described in this study might contribute to improve *O. maya* welfare during laboratory practices, as well as the quality of research data.

## Data Availability Statement

The datasets generated for this study are available on request to the corresponding author.

## Ethics Statement

The animal study was reviewed and approved by Animal Ethics Committee of the Faculty of Chemistry at Universidad Nacional Autónoma de México (UNAM, Sisal). Permit Number: Oficio/FQ/CICUAL/099/15.

## Author Contributions

KR and CR conceived and designed the study, and drafted the manuscript. KR, MA, and JP conducted the experiments. CR and CP performed the data analysis. All authors interpreted the findings together and approved the final version of the manuscript.

## Conflict of Interest

The authors declare that the research was conducted in the absence of any commercial or financial relationships that could be construed as a potential conflict of interest.

## Footnotes

1 https://www.nc3rs.org.uk/arrive-guidelines

## Supplementary Material

FIGURE S1 Daily Growth Coefficient (DGC, %BW d^–1^) of *Octopus maya* juveniles exposed or not to the anesthetic agents. (SW − H; SW + H). SW: sea water (25°C, unless if specified); SW − H: sea water, without handling; SW + H: sea water, with handling (see text for details). EtOH, ethanol; MgCl_2_, magnesium chloride; Mix, ethanol in combination with magnesium chloride (concentrations are provided in this order).

FIGURE S2 Plastic containers for housing *Octopus maya* juveniles (not to scale).

TABLE S1 Changes on wet weight (BW, ww, g) and daily growth coefficient (DGC; %BW d^–1^) of *Octopus maya* juveniles exposed or not to the different substances (or cold sea water) and handling. Values as mean ± SD (g). Different letters mean statistical differences between row data at *p* < 0.05 level. SW: sea water (25°C, unless if specified); SW − H: sea water, without handling; SW + H: sea water, with handling (see text for details). EtOH, ethanol; MgCl_2_, magnesium chloride; Mix, ethanol in combination with magnesium chloride.

TABLE S2 ANCOVA analysis of the relationship between wet weight and experimental time of *Octopus maya* juveniles exposed or not to the different substances or cold sea water and handling. Note that two groups were formed: one of the animals anesthetized with clove oil and manipulated and the other with the remaining treatments.
