# Corrigendum: Short and Long-Term Effects of Anesthesia in *Octopus maya* (Cephalopoda, Octopodidae) Juveniles

**DOI:** 10.3389/fphys.2020.599873

**Published:** 2020-10-07

**Authors:** Katina Roumbedakis, Marina N. Alexandre, José A. Puch, Maurício L. Martins, Cristina Pascual, Carlos Rosas

**Affiliations:** ^1^AQUOS – Aquatic Organisms Health Laboratory, Department of Aquaculture, Federal University of Santa Catarina (UFSC), Florianopolis, Brazil; ^2^Unidad Mulidisicplinaria de Docencia e Investigación, Facultad de Ciencias, Universdidad Nacional Autónoma de México (UNAM), Mexico City, Mexico

**Keywords:** anesthetic, cephalopods, oxygen consumption, criteria, growth, mortality, animal welfare

In the original article, there was an error in the Acknowledgments statement.

A correction has been made to ***Acknowledgments Statement***. It should read as follow:

“We would like to thank our colleagues from the UNAM (Mexico), specially Claudia Camaal, Elisa Chan, and Ariadna Sánchez for their technical collaboration. We are grateful for the substantial contributions made by Prof. Paul Andrews (Associate Fellow, SZN) during various steps of this manuscript. KR is grateful to the Coordination for the Improvement of Higher Education Personnel (CAPES, Brazil), the Ministry of Foreign Affairs and International Cooperation (MAECI, Italy) and the Association for Cephalopod Research CephRes—a non-profit organization (Italy). UNAM and CephRes supported the costs of this publication. This work is considered a contribution to the activities of the COST Action FA1301 Cephs*In*Action A network for improvement of cephalopod welfare and husbandry in research, aquaculture and fisheries http://www.cephsinaction.org/.”

The authors apologize for this error and state that this does not change the scientific conclusions of the article in any way. The original article has been updated.

