# Supplementary figures and images for: Short and Long-Term Effects of Anesthesia in Octopus maya (Cephalopoda, Octopodidae) Juveniles
Source: Front Physiol. 2020 Jun 30;11:697. doi: 10.3389/fphys.2020.00697 (PMC7338579; doi:10.3389/fphys.2020.00697)

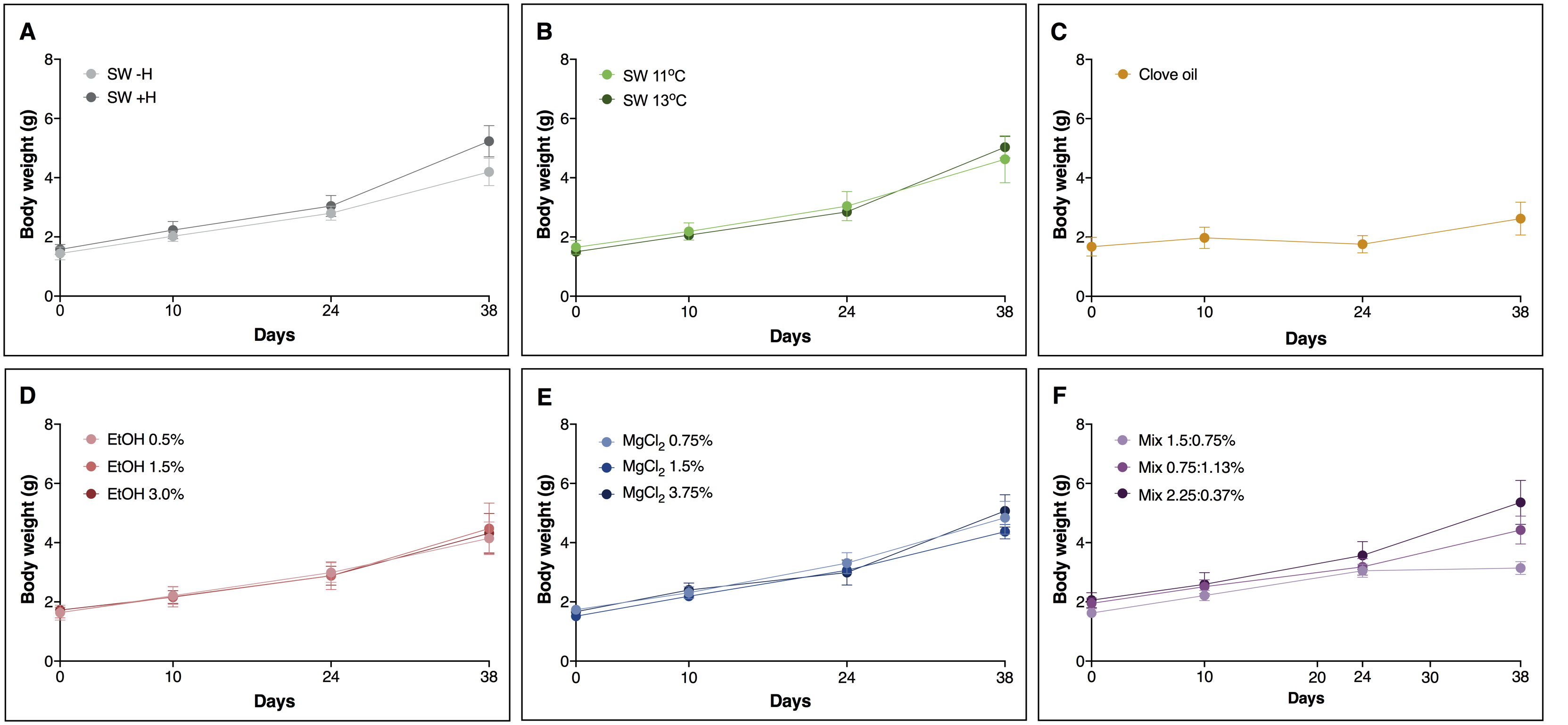

Supplement: FIGURE S1 — Daily Growth Coefficient (DGC, %BW d–1) of Octopus maya juveniles exposed or not to the anesthetic agents. (SW − H; SW + H). SW: sea water (25°C, unless if specified); SW − H: sea water, without handling; SW + H: sea water, with handling (see text for details). EtOH, ethanol; MgCl2, magnesium chloride; Mix, ethanol in combination with magnesium chloride (concentrations are provided in this order). [file Image_1.TIFF]

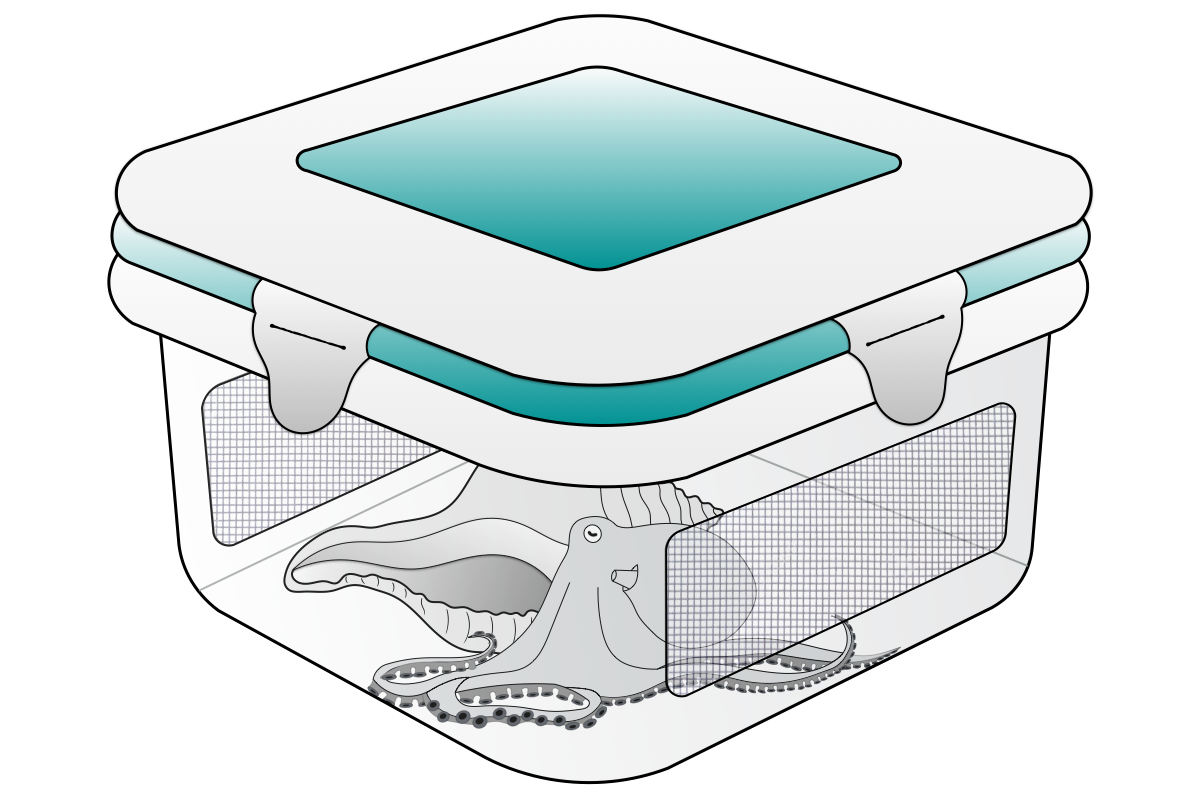

Supplement: FIGURE S2 — Plastic containers for housing Octopus maya juveniles (not to scale). [file Image_2.TIFF]
