# Supplementary material for: Short and Long-Term Effects of Anesthesia in Octopus maya (Cephalopoda, Octopodidae) Juveniles
Source: Front Physiol. 2020 Jun 30;11:697. doi: 10.3389/fphys.2020.00697 (PMC7338579; doi:10.3389/fphys.2020.00697)
Supplement: TABLE S1 — Changes on wet weight (BW, ww, g) and daily growth coefficient (DGC; %BW d–1) of Octopus maya juveniles exposed or not to the different substances (or cold sea water) and handling. Values as mean ± SD (g). Different letters mean statistical differences between row data at p < 0.05 level. SW: sea water (25°C, unless if specified); SW − H: sea water, without handling; SW + H: sea water, with handling (see text for details). EtOH, ethanol; MgCl2, magnesium chloride; Mix, ethanol in combination with magnesium chloride. [file Table_1.pdf]

TABLE S1 | Changes on wet weight (BW, ww, g) and daily growth coefficient (DGC; %BW d^{-1}) of *Octopus maya* juveniles exposed or not to the different substances (or cold sea water) and handling. Values as mean ± SD (g). Different letters mean statistical differences between row data at $p < 0.05$ level. SW: sea water (25ºC, unless if specified); SW –H: sea water, without handling; SW +H: sea water, with handling (see text for details); EtOH: ethanol; $MgCl_2$: magnesium chloride; Mix: ethanol in combination with magnesium chloride.

	SW -H	SW +H	SW 11°C	SW 13°C	EtOH 0.5%	EtOH 1.5%	EtOH 3.0%	$MgCl_2$ 0.75%	$MgCl_2$ 1.5%	$MgCl_2$ 3.75%	Mix 1.5:0.75%	Mix 0.75:1.13%	Mix 2.25:0.37%	Clove oil
Day 0														
BW, ww, g	1.58 a	1.44 a	1.65 a	1.50 a	1.64 a	1.64 a	1.73 a	1.73 a	1.52 a	1.67 a	1.62 a	1.95 a	2.06 a	1.68 a
SD	0.44	0.57	0.62	0.29	0.67	0.48	0.32	0.42	0.30	0.43	0.26	0.47	0.67	0.84
N	7	7	7	7	7	7	7	7	7	7	7	7	7	7
Day 10														
BW, ww, g	2.23	2.13	2.19	2.06	2.21	2.18	2.16	2.31	2.19	2.40	2.21	2.51	2.59	1.97
SD	0.78	0.62	0.77	0.36	0.78	0.91	0.57	0.53	0.27	0.62	0.45	0.61	1.05	0.95
N	7	7	7	7	7	7	7	7	7	7	7	7	7	7
Day 24														
BW, ww, g	3.04	2.80	3.04	2.85	2.99	2.88	2.88	3.31	3.07	2.99	3.05	3.18	3.57	1.76
SD	0.94	0.63	1.30	0.39	0.88	1.23	0.84	0.93	0.44	0.85	0.54	0.74	1.24	0.66
N	7	7	7	6	7	7	7	7	7	4	6	7	7	5
Day 38														
BW, ww, g	5.23	4.67	4.63	5.03	4.15	4.48	4.32	4.84	4.37	5.07	4.47	4.43	5.36	2.62
SD	1.18	1.22	1.77	0.89	1.24	2.10	1.63	1.46	0.60	1.10	0.55	1.24	1.96	1.24
N	5	7	5	6	5	6	6	7	6	4	5	7	7	5
Day 0 to 10														
DGC, %BW d^{-1}	3.47	3.89	2.80	3.18	3.00	2.84	2.26	2.87	3.68	3.66	3.10	2.53	2.31	1.63
SD	1.22	1.37	1.48	1.25	0.72	1.35	1.44	0.95	1.05	1.43	1.18	1.00	3.24	1.14
N	7	7	7	7	7	7	7	7	7	7	7	7	7	7
Day 10 to 24														
DGC, %BW d^{-1}	2.22	1.96	2.36	2.31	2.17	2.01	2.06	2.56	2.40	1.57	2.29	1.69	2.29	-0.83
SD	0.98	0.87	1.02	3.05	0.74	0.53	1.56	0.87	0.64	5.18	2.22	0.68	2.17	4.73
N	7	7	7	7	7	7	7	7	7	7	7	7	7	7
Day 24 to 38														
DGC, %BW d^{-1}	3.87	3.65	3.00	4.06	2.33	3.15	2.89	2.72	2.53	3.77	2.73	2.35	2.90	2.86
SD	0.97	0.92	2.32	0.72	0.52	1.05	0.39	0.32	1.01	5.48	0.55	0.90	1.05	1.17
N	7	7	5	6	5	6	6	7	6	4	5	7	7	5
Day 0 to 38														
DGC, %BW d^{-1}	3.15 b	3.09 b	2.71 b	3.18 b	2.45 b	2.65 b	2.41 b	2.70 b	2.79 b	2.93 b	2.66 b	2.15 b	2.52 b	1.18 a
SD	0.49	0.48	1.11	0.58	0.62	0.72	0.68	0.41	0.51	0.22	0.48	0.59	0.59	1.16
N	5	7	5	6	5	6	6	7	6	4	5	7	7	5
