# Supplementary material for: Short and Long-Term Effects of Anesthesia in Octopus maya (Cephalopoda, Octopodidae) Juveniles
Source: Front Physiol. 2020 Jun 30;11:697. doi: 10.3389/fphys.2020.00697 (PMC7338579; doi:10.3389/fphys.2020.00697)
Supplement: TABLE S2 — ANCOVA analysis of the relationship between wet weight and experimental time of Octopus maya juveniles exposed or not to the different substances or cold sea water and handling. Note that two groups were formed: one of the animals anesthetized with clove oil and manipulated and the other with the remaining treatments. [file Table_2.pdf]

TABLE S2 | ANCOVA analysis of the relationship between wet weight and experimental time of *Octopus maya* juveniles exposed or not to the different substances (or cold sea water) and handling. Note that two groups were formed: one of the animals anesthetized with clove oil and manipulated and the other with the remaining treatments.

Statistics	Animals exposed or not to the different substances or cold sea water (except clove oil) ± handling	Animals exposed to clove oil + handling
Slope	0.028 ± 0.0020	0.016 ± 0.0038
Y-intercept when X= 0.0	0.47 ± 0.045	0.26 ± 0.080
X-intercept when Y= 0.0	-17	-16
1/slope	35	62
95% interval of confidence		
slope	0.024 to 0.032	0,0083 to 0,024
Y-intercept when X=0.0	0.38 to 0.56	0.089 to 0.42
X-intercept when Y=0.0	-23 to -12	-47 to -4.0
Goodness of Fit		
R square	0.79	0.45
Sy.x	0.21	0.26
Is slope significantly non-zero?		
F	195	18
DFn, DFd	1,0, 52	1,0, 22
	< 0.0001	0.0003
	Significant	Significant

Are the slopes equal? No ;

F = 12.8292. DFn=1 DFd=74

P=0.0006077
